# Joining Inventory by Parataxonomists with DNA Barcoding of a Large Complex Tropical Conserved Wildland in Northwestern Costa Rica

**DOI:** 10.1371/journal.pone.0018123

**Published:** 2011-08-16

**Authors:** Daniel H. Janzen, Winnie Hallwachs

**Affiliations:** Department of Biology, University of Pennsylvania, Philadelphia, Pennsylvania, United States of America; Centre National de la Recherche Scientifique, France

## Abstract

**Background:**

The many components of conservation through biodiversity development of a large complex tropical wildland, Area de Conservacion Guanacaste (ACG), thrive on knowing what is its biodiversity and natural history. For 32 years a growing team of Costa Rican parataxonomists has conducted biodiversity inventory of ACG caterpillars, their food plants, and their parasitoids. In 2003, DNA barcoding was added to the inventory process.

**Methodology/Principal Findings:**

We describe some of the salient consequences for the parataxonomists of barcoding becoming part of a field biodiversity inventory process that has centuries of tradition. From the barcoding results, the parataxonomists, as well as other downstream users, gain a more fine-scale and greater understanding of the specimens they find, rear, photograph, database and deliver. The parataxonomists also need to adjust to collecting more specimens of what appear to be the “same species” – cryptic species that cannot be distinguished by eye or even food plant alone – while having to work with the name changes and taxonomic uncertainty that comes with discovering that what looked like one species may be many.

**Conclusions/Significance:**

These career parataxonomists, despite their lack of formal higher education, have proven very capable of absorbing and working around the additional complexity and requirements for accuracy and detail that are generated by adding barcoding to the field base of the ACG inventory. In the process, they have also gained a greater understanding of the fine details of phylogeny, relatedness, evolution, and species-packing in their own tropical complex ecosytems. There is no reason to view DNA barcoding as incompatible in any way with tropical biodiversity inventory as conducted by parataxonomists. Their year-round on-site inventory effort lends itself well to the sampling patterns and sample sizes needed to build a thorough barcode library. Furthermore, the biological understanding that comes with barcoding increases the scientific penetrance of biodiversity information, DNA understanding, evolution, and ecology into the communities in which the parataxonomists and their families are resident.

## Introduction

**Figure 1:**
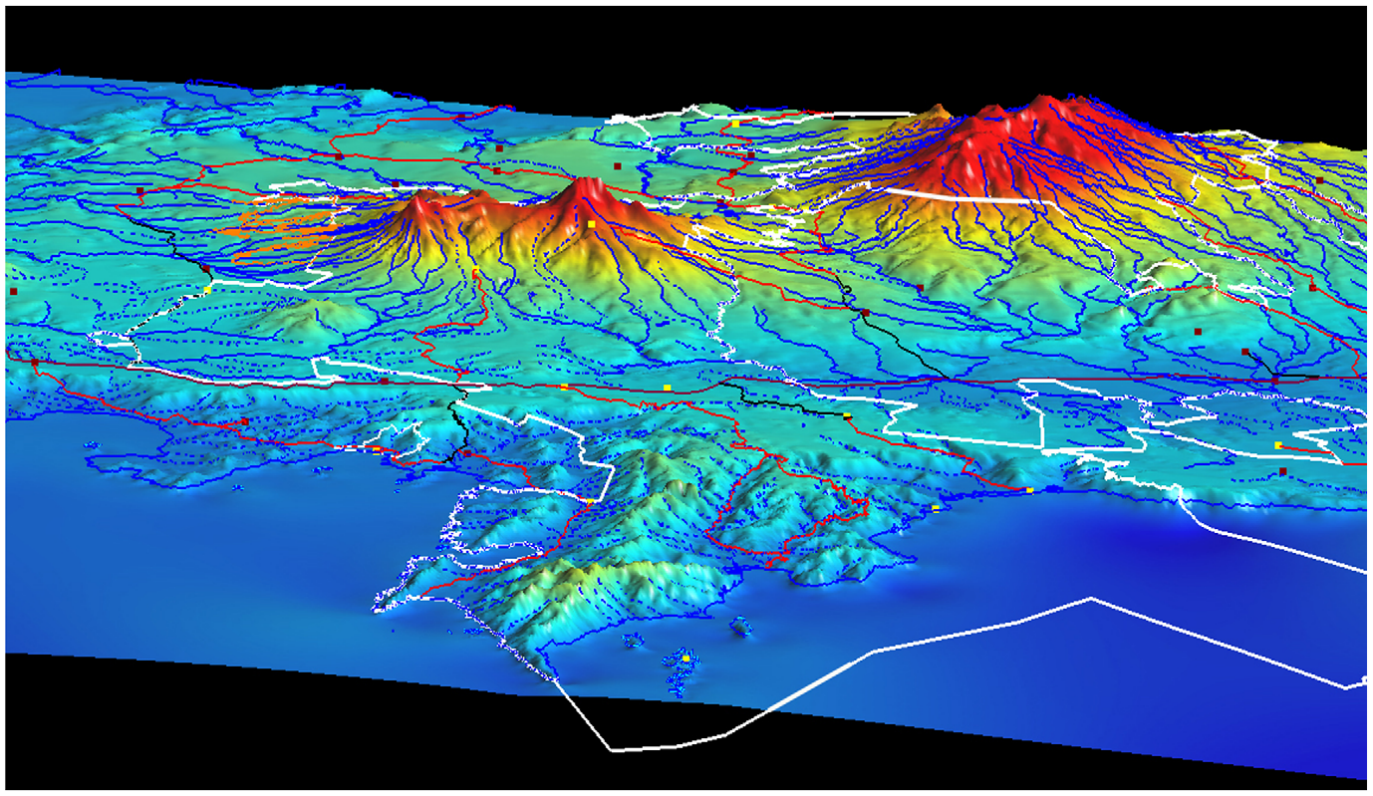
A 3D map (2008) of 1,630 km^2^ Area de Conservacion Guanacaste (ACG) as it is viewed from over the Pacific Ocean. ACG is all the area inside the white outline (and inside the small orange-outlined area that is Sector Del Oro). Dry forest covers the Pacific lowlands, cloud forest covers the volcano tops (red), and rain forest covers the background lowlands; intergrades are yellowish on the slopes. Volcán Orosí (1,450 m) is on the far left, Volcán Cacao (1,695 m) is in the center, and the complex of Volcán Rincón de la Vieja, Volcán Von Seebach (1,895 m) and Volcán Santa Maria (1,916 m) is on the right. Yellow filled squares are ACG biological and administrative stations, red filled squares are some of the schools serviced by the ACG Programa de Educación Biológica. The Interamerican Highway (Pan-American Highway of old) passes horizontally through the center of the image, the coastal town of La Cruz is out of sight to the left, the town of Liberia is out of sight to the right, and Nicaragua is barely out of sight to the left. The uppermost central yellow square is Estación Biológica Caribe and the central yellow square at the end of the black road is Estación Biológica Santa Rosa. Image credit, Waldy Medina.

The terrestrial 1,200 km^2^ of Area de Conservacion Guanacaste (ACG) in northwestern Costa Rica encompasses dry forest, cloud forest (to 2000 m elevation), and rain forest, and all their intergrades, extending 80 km from the Pacific coast to the Caribbean rainforest lowlands at 70 m elevation (http://janzen.sas.upenn.edu/saveit.html) [1]–[3]. This single area of highly contoured terrain (Figure 1) is covered by a mosaic of ages and kinds of natural regeneration ranging from old growth on a great variety of soil types, exposures and rainfall patterns, to contemporary 1–400-year-old regeneration in old pastures, fields, hunting grounds, fishing areas, house sites, roads, and other anthropogenic disturbances [1], [4]–[6].

**Figure 2:**
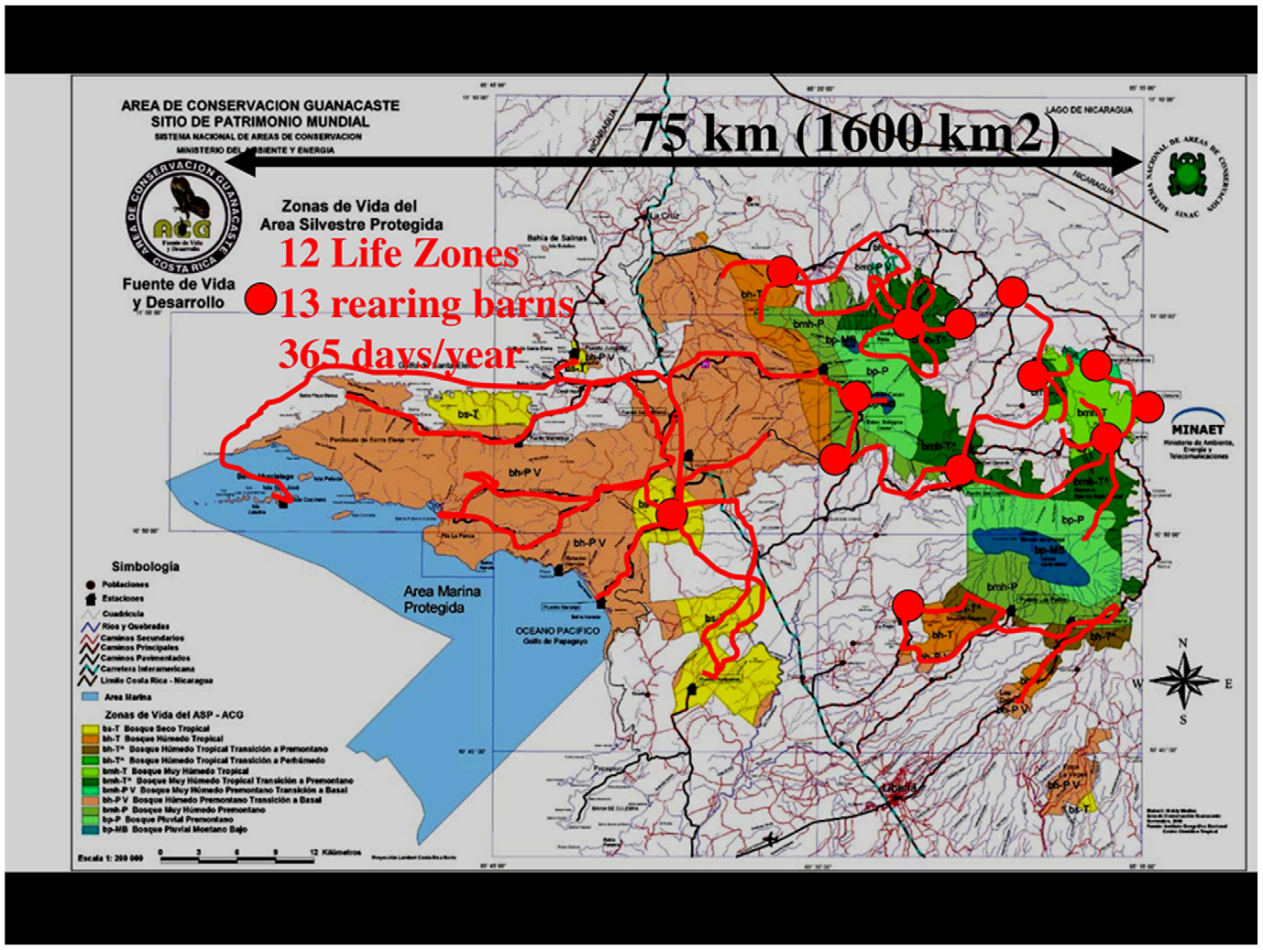
This map outlines Area de Conservacion Guanacaste (ACG) Life Zones as overlain with caterpillar inventory rearing barns (red circles) and general transit access roads and trails (red lines). The 12 Life Zones cover, starting from the left, marine (light blue) and dry forest (browns and yellows) to the upper elevation cloud forest (dark blue) and rain forest (various greens), with the expected intergrades. (2010). Image credit, Waldy Medina and Daniel Janzen.

As a conserved and restoring tropical wildland, ACG is overlain with a webbing of old and new roads, trails and living-working sites (Figure 2). Among these are 13 caterpillar rearing barns at ACG biological (and administrative) stations, each used by 1–5 Costa Rican parataxonomists living in or adjacent to ACG to carry out their microgeographically-based portion of the on-going inventory of the caterpillars, and their food plants and parasitoids, for all of ACG [3]. This inventory began in 1978, is planned to continue until “complete” for all ACG Lepidoptera taxa except for leaf miners, and currently has “inventoried” at least 9,000 of the estimated 15,000 ACG species (e.g., http://janzen.sas.upenn.edu). This estimate of the total Lepidoptera biodiversity is based on 30-plus years of light trapping ACG and other parts of Costa Rica by the senior authors and INBio, Costa Rica's Instituto Nacional de Biodiversidad. For technical reasons, the inventory does not currently attempt to include leaf-mining caterpillars.

By “inventory” is meant a) find that species of caterpillar in nature on at least one of its food plants, and collect it with food plant foliage to bring back to the rearing barn, b) photographically document its form/colors, c) verify that it is eating that plant, d) rear it through to a taxonomically tractable adult (this is often a long and patience-requiring process of supplying fresh food of the same species every 3–5 days until the caterpillar pupates, then checking every pupa daily to find freshly-emerged adults before they damage themselves by flying in their rearing container), e) find and rear more conspecifics for at least a portion of its range of food plants and naturally occurring parasitoids, f) carry out the taxonomic identification/clarification of the caterpillar-plants-parasitoids to the degree possible, g) database all of this process and information as it is being gathered, h) voucher all specimens to the degree logistically and intellectually possible, i) make all of this information freely and publically available on the web (e.g., http://janzen.sas.upenn.edu, http://butterfliesofamerica.com/), k) where time, funding, motivation, and energy permits, generate published syntheses, summaries and/or question-specific analyses, and l) deposit all vouchers in large public museums while continuing to collaborate with the taxasphere to clarify them taxonomically while retroactively incorporating this information into primary databases such as at http://janzen.sas.upenn.edu and http://www.boldsystems.org/views/login.php, and derivative databases such as GenBank and GBIF.

All of this effort has been, and is being today, carried out by a dynamic large network of mostly urban taxonomists and their institutions, in coordination with the daily work of a (today) 33-member team of field-based and rural-living, Costa Rican ACG career parataxonomists [7]–[9], [10]–[12] (see acknowledgments for a list), 4-member INBio team of curators/taxonomists (Isidro Chacon, Bernardo Espinoza, Jenny Phillips, Ronald Zuñiga), and the field coordinator, Felipe Chavarria. This integration and coordination is variously conducted full-time by us at the University of Pennsylvania and in ACG while visiting the 13 biological stations, by the taxonomists and parataxonomists themselves, and by a large and frequently changing network of auxiliary volunteer integrators and supporters.

The place - the ecological and sociological setting - is ACG and its 15,000 species of caterpillars living in the large restoring (and old growth) protected expanses of its three major forested tropical ecosystems. The parataxonomists are the source of the original data and voucher specimens. The taxasphere, meaning the collective whole of taxonomists, their literature, their specimens, their websites, and their institutions [13], offers the information networking and reference vocabulary, and its integration, that is frequently generated by taxonomic phylogenetic inference from evolution-based taxonomy. Information management coupled with modern electronics provides the storage and dissemination of this mass of initially amorphous and highly particulate information offered to the world at large, with Filemaker Pro, Exel, GIS, .jpg, .pdf, Apple, Google fusion tables, and the Internet, currently being the primary protocols.

Out of this huge socio-biological ecosystem, we focus here on some of the activities of the parataxonomists with respect to the addition of DNA barcoding to the entire ACG caterpillar-parasitoid-plant inventory, beginning in 2003 [3], [14]–[17].

### Parataxonomists

“Parataxonomist” as used here and throughout the conservation and development history of ACG means a person derived from the rural work force who has been on-the-job trained, facilitated, and stimulated to be able to carry out the same performance of biodiversity inventory in the field as could/would a graduate student or post-doc in taxonomy/ecology. Their career is to find and field-document “everything” in this or that portion of an ecosystem, while being resident in or near ACG [8]–[12], [14].

**Figure 3:**
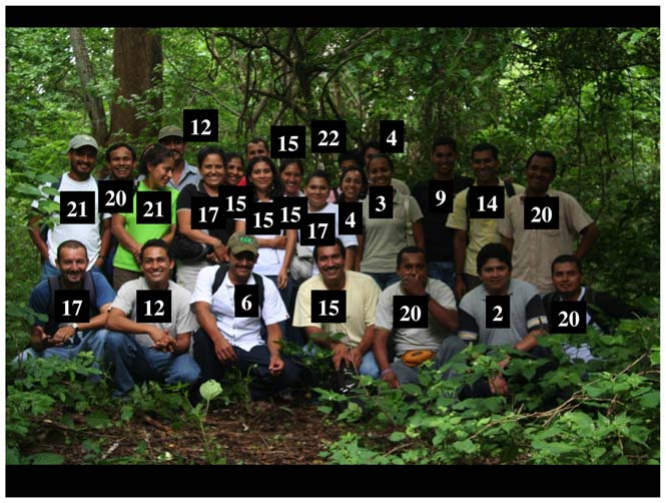
This is the majority of the members of the ACG Costa Rican resident parataxonomist team (a.k.a “gusaneros”) in 2008 (Estación Biológica Santa Rosa). Each is labeled with the number of years he or she has worked conducting the ACG caterpillar inventory, and adult moth and butterfly inventory (BioLep). Image credit, Daniel Janzen.

Parataxonomists are thus resident long-term employees (Figure 3, [18]–[19], and http://janzen.sas.upenn.edu/caterpillars/methodology/how/inventorymeth.html), with grade school to high school formal education, young to middle-aged adults, often married with children, and not aimed professionally at “escape” to large urban centers and “higher” education or administrative positions (though some do this). A parataxonomist may focus on inventory of a particular taxon or place as part of some larger plan of inventory or taxonomic thoroughness. The label was borrowed from the word “paramedic”. It was chosen to encompass the paramedics' jack-of all-trades facilitation of the work of a more intensely trained specialist higher up on the information chain, and while working in a more ever-present manner than can ever be expected of the (ever decreasing) number of thinly distributed (and expensive) specialists - be they neurosurgeons or the only world-level specialists on the taxonomy of Ichneumonidae or Choreutidae, and who used much of a lifetime to get there.

A particular parataxonomist may be on the government payroll of ACG as a civil servant, or in a NGO research project supported by “outside” funding, or both. Equally, his or her working circumstances are likely to be funded by an unholy mix of resources. But regardless of who pays the bill, the goal is capture of biodiversity data in the field and facilitate the flow of that information down the processing chain to a very wide variety of users. The users may be ACG in-house actions such as a) direct conservation decisions for land purchase, b) raw material for education programs, c) granting permits for outside researchers to do destructive sampling, d) monitoring restoration and recovery of ACG species, including climate change impacts, and/or e) site preparation for the integration of industrial-level disturbance with a conserved wildland. As such they are integral members of the biodiversity management and development team required by a large complex conserved wildland [20]–[23]. And/or they may be conducting very outwardly-focused actions, funded by external sources, such as providing the specimens and associated collateral that are the raw materials of the taxasphere in general, and of specific initiatives such as the biodiversity inventory of ACG, and iBOL (a mission to DNA barcode the world's biodiversity [24]; http://ibol.org). Parataxonomists can therefore be a major ingredient in the platform of understanding complex wild tropical ecosystems, and especially in biodiversity development of them for their integration into society.

Parataxonomists originated in the temporarily employed “field assistants” and “collectors” of taxonomic expeditions in centuries past, and grade into the occasional resident individual who even made a long-term living providing (usually) tropical specimens to a network of developed-country museums. Even today, there is a tendency among some parts of the academic community to continue to call them “local technicians”. However, “technician” seriously understates the level of responsibility and initiative developed by most parataxonomists. Initially, we attempted to categorize them by their activities into “paraecologists” and “parataxonomists” but found the distinction to be confusing to them and to the community of users of their information, so have remained with parataxonomist as a convenient descriptive term. We refrain from entering into debate with those who wish to define or evaluate parataxonomists by other yardsticks (often) in order to depreciate the concept of introducing diverse and intellectually interesting employment into the rural workforce, for both the improved health of the agroecosystem and its integration with wildland areas conserved as such.

### DNA barcoding

Since 2003, the ACG caterpillar-plant-parasitoid inventory has been both a proof-of-principle (a.k.a. “lab rat for biodiversity development”), and a major on-the-job user, of DNA barcoding (the use of a short standardized segment of DNA for specimen identification and species discovery). The history is described in detail elsewhere [3], [14]–[16], [25]–[26].

Here we describe a few of the core processes of this 7-year effort to integrate DNA barcoding with the ACG caterpillar-food plant-parasitoid inventory, and therefore with conservation of ACG through its internal and external biodiversity development.

### 1) Voucher specimens as base for a DNA barcode

While the parataxonomists' activities have always been central to ACG inventory, in 2003-onwards the large backlog of museum-based inventory vouchers became a major database resource for road-testing barcoding, and the intricacies of taxonomic biodiversity discovered via ACG barcoding has greatly increased the volume of voucher specimens, with a subsequent large impact on museum storage space and curation needs.

**Figure 4:**
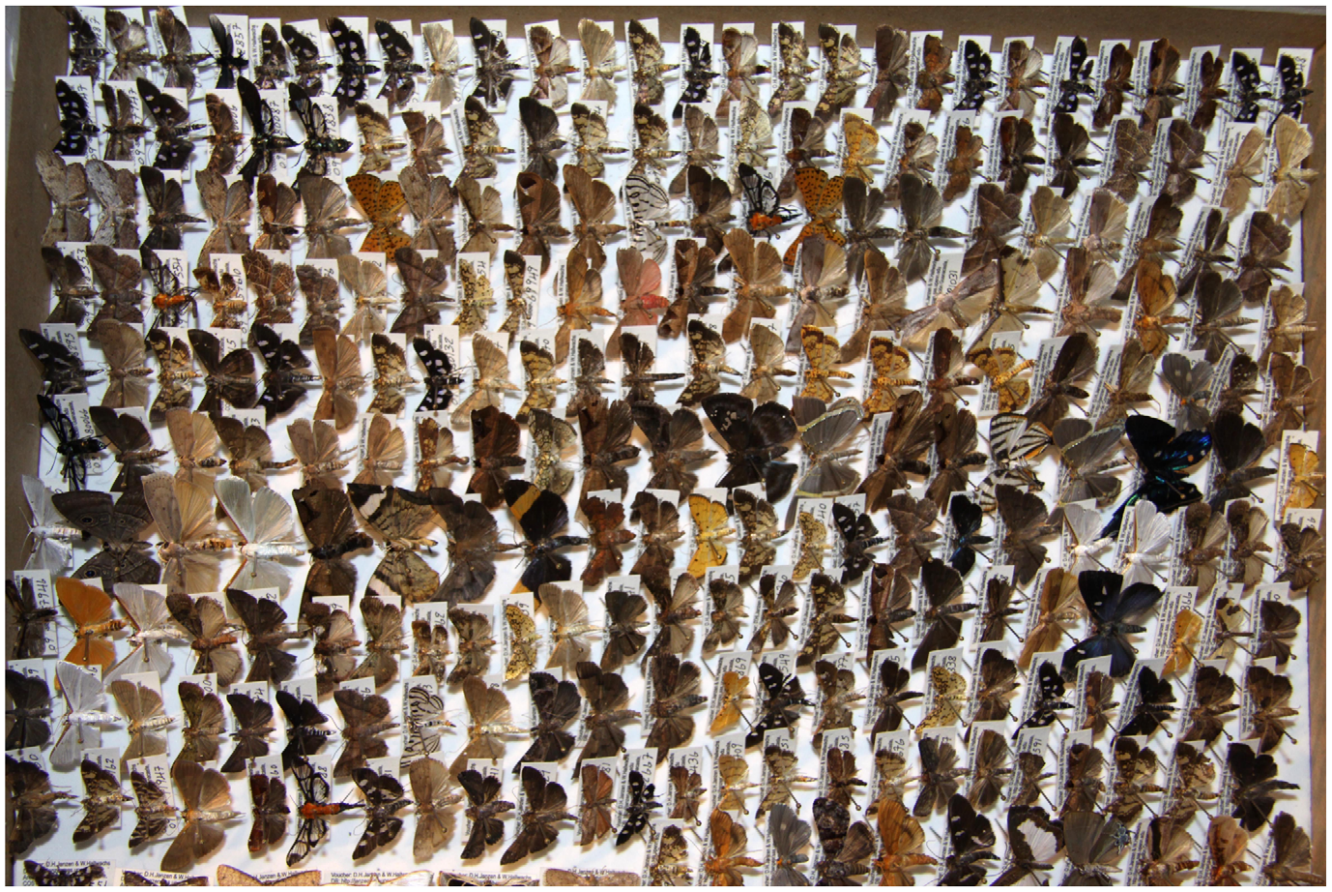
This is a box (same size as the white box in **Figure 8**) of reared, databased, spread and oven-dried ACG small moths and butterflies, in the form that they were delivered by a parataxonomist (Johan Vargas) to the Santa Rosa clearing center. Each specimen has its unique voucher code on its pin, and each will lose one leg to the barcoding process by Tanya Dapkey as it passes through the central clearing center at the University of Pennsylvania on its way to permanent residence in the Smithsonian Institution, INBio, Canadian National Collection, or other museum. (August 2010). Image credit, Daniel Janzen.

Since its inception in 1978, the ACG caterpillar-food plant-parasitoid inventory has emphatically saved voucher specimens (e.g., Figure 4) of “everything” to a) provide raw material for the taxasphere to examine and/or dissect, b) ensure that the photographed caterpillars (soft body, impossible to preserve realistically) correctly bear the name assigned to their adults, c) conduct the many years of taxonomic work to identify or describe the species treated, and d) be available for further morphological/functional studies of these (often once-in-a-lifetime, often irreplaceable) specimens harvested at such a high cost in dollars and human resources. The care and quality control of voucher specimens has always been a central point of parataxonomist training, feedback, and evolution of methods (including the key principle that reporting errors is actively encouraged).

DNA barcoding, both as tool-development and inventory quality control/accuracy in an inventory, is an outstanding example of d) above. Essentially all of these inventory vouchers – now numbering at least a half million museum specimens dried or in ethanol - were collected, prepared and databased, along with their collateral, by the ACG parataxonomists. The specimens are now housed in, or *en route* to, a network of 8 major public museums. *There is no way that such a mass of barcodeable specimens and collateral could have been accumulated by the authors and the occasional academic visitor/collector working alone in the standard expeditionary protocol*, a protocol to which Costa Rican biodiversity has been subject for several centuries [27]. Among the very first acts of integrating DNA barcoding into the ACG inventory, we began in 2003 (as much as technically and logistically possible) to DNA barcode the mass of these “older” voucher specimens, while simultaneously initiating the routine barcoding of the new “fresh” incoming vouchers. Paul Hebert, Alex Smith, Mehrdad Hajibabaei and others on the laboratory team at the Biodiversity Institute of Ontario (BIO), University of Guelph, performed the technical gyrations of sequencing this mix of relatively old and fresh specimens, and used them for age-based comparative sequencing as well.

In other words, because we have had the team of parataxonomists investing a huge amount of intellectual and physical labor vouchering the inventory specimens, a massive resource of databased specimens (and collateral data) was available to the DNA barcoding initiative from the outset. This allowed the examination of questions of sample sizes, ages of specimens, lengths of barcodes, correlations of non-barcoding morphology-based identifications with barcoding results, correlations of barcodes with microgeography and ecology, etc., and all from “one place”. Furthermore, the directionality of the ongoing inventory can be adjusted at any time to further examine a puzzle suggested by the barcoding of thes older specimens.

The 600-plus specimens of the seemingly single species “*Astraptes fulgerator azul*” (a skipper butterfly in the family Hesperiidae that ranges from Texas to South America) are outstanding examples. They were reared by the ACG parataxonomists prior to barcoding and saved because the enormous variety of plants they fed on in nature far surpassed that of other skipper butterflies. And they filled 18 drawers in the National Museum of Natural History in Washington, D.C. They were then found by the combination of morphological inspection, food-plant correlation, microgeographic distribution within ACG, and DNA barcoding, to comprise at least 10 sympatric and excruciatingly similar species of butterflies [26]. This example has led to further rearing and barcoding by the parataxonomists in search of yet more species hidden within “*A. fulgerator*” (one more, “*Astraptes* ENT”, has been located by the inventory to date), and therefore has led to yet more vouchers to be deposited in the USNM. This has swelled the collection, but also this barcoding has yielded the tools and raw data to a) explore the ability of these sibling species to ecologically probe each other's food plants, b) genetically probe each other, c) understand and watch the microgeographic overlaps of their complex ranges, and d) even attempt to extend the effort to other countries. The parataxonomists who found and reared the caterpillars initially are well placed in geography, experience and understanding to carry out as much of this extension as the budget permits.

However, it does need to be emphasized that this ACG barcoding effort in turn has only been possible because the world's biodiversity museums have been generous with their space allocation to the this large and growing collection of, to some degree, “non-taxonomic” voucher specimens. In 2003, the Costa Rican Hesperiidae occupied about 30 drawers in the USNM, and today they occupy about 220 drawers (of the most expensive real estate in downtown Washington, D.C.). This expansion is at least 80% due to the parataxonomists being focused on locating and rearing wild Hesperiidae caterpillars as a target for barcode exploration. In general, taxonomic collections have emphasized using their scarce space and human resources for geographic as well as taxonomic breadth, rather than intense sampling from one place and the study of intraspecific variation. While global DNA barcoding will eventually create geographic breadth and thoroughness, in these initial exploratory stages, large samples of barcoded vouchers from one place have other values as well, even if they swamp museum available space with what appears to be taxonomically redundant material. This in turn means that in addition to an increased need for more financial resources for the field side of DNA barcoding by parataxonomists and others, more resources are also needed to cover the costs of the voucher storage and curation to backstop a high-quality DNA barcode reference library based on barcode vouchers. Incidentally, this sudden and long unanticipated use of museum specimens for barcoding is a very nice example of how the bug on the pin is far more than a taxonomic tool - it is a black box of (currently unappreciated) information that may be gradually opened as the technologies for capturing its contained information come on line.

### 2) Reared vouchers and their collateral information

Because the ACG parataxonomists are rearing the barcoded adult vouchers from caterpillars, the inventory provides extra layers of collateral data (food plants, parasitoids, trophic lineages, microgeography). DNA barcoding originated in the taxasphere [14]–[15], [26]. The taxasphere, much as it appreciates and wants natural history collateral associated with the specimens, is accustomed to carrying out the great bulk of its work with museum specimens that have at best only locality, date, and collector/collection information for adults as collateral. As such, those data and adult morphology are all they have available to correlate with barcoding results. The three-way correlation between the three sets of information – morphology, caterpillar ecology, and barcodes – allows far greater taxonomic and biological resolution and data checking for any one of the three than would be the case with only one or two of them. This in turn has allowed the inventory to pursue the taxonomic significance of much finer differences between and among barcode clusters than is generally the case with standard museum specimens that are lacking potentially species-specific and specimen-specific ecological data.

For example, the parataxonomists have reared thousands of parasitoid wasp specimens that appeared to be the morphologically-defined species *Apanteles leucostigmus* (Braconidae, Microgastrinae). *A. leucostigmus* is 2–3 mm long and black with a white stigma in the wing. The wasps were reared from 40-plus species of wild-caught ACG Hesperiidae caterpillars over the first 25 years of the inventory, and it was easy to conclude that this species of wasp is a host-specialist on caterpillars of Hesperiidae, but a host-generalist within Hesperiidae. However, when specimens from 1,000-plus rearings of this wasp (stored as refrigerated vouchers in ethanol in Jim Whitfield's cold room at the University of Illinois) were barcoded, they were found to comprise at least 37 distinct lumps of DNA barcodes in a standard NJ tree [28]. Is this then a 37-morph species? When the barcodes were correlated with the caterpillar and food-plant records recorded and databased by the parataxonomists over all these years, it became immediately obvious that “*Apanteles leucostigmus*” is not a generalist among Hesperiidae caterpillars, but rather, a large (and still growing through further ACG inventory) complex of extreme specialists, each specialized at using the caterpillars of its particular species, or morphologically closely related species, of skipper butterfly (and see [29]–[30] for parallel examples among Tachinidae fly parasitoids of ACG caterpillars).

At the other end of the scale, the barcode probing of large samples of vouchers accumulated by the parataxonomists over many years across many species of food plants, from what appear to be essentially identical caterpillars producing what appear to be identical adult moths, has also confirmed that there really are some quite amazing generalists in these ecosystems that are so rich in specialists [28]–[30]. “*Anacrusis nephrodes*” and “*Anacrusis aulaeodes*” are a recently discovered example. They are medium-large totally sympatric rain forest Tortricidae moths that have green undistinctive caterpillars (except that they are microsnake mimics [31]) that live solitarily in an undistinctive irregular mass of silk, turds and tangled leaves. They are found occasionally by the inventory. *A. nephrodes* has been reared from more than 200 species of plants in more than 50 families, and *A. aulaeodes* about half that (e.g., query food plant records for *Anacrusis nephrodes* at http://janzen.bio.upenn.edu/caterpillars/database.lasso). Both species are rain forest generalists by any yardstick. But are they? Because the parataxonomists have accumulated a very large sample of more than 1,000 reared vouchers over 20 years, it was possible to initially submit 30 specimens of each moth species for barcoding (to get the basic barcode and to ask if they were truly generalist, as was the case with a Papua-New Guinea tortricid caterpillar [32]). This generated the frustrating result of “yes, fragmentation into groups of barcodes (suggesting cryptic species) but also a very high proportion of sequencing failures”. The latter then turned out to be sequence-level interference by a very high proportion of *Wohlbachia* infections of the adult moths. However, once the “correct” primer was encountered that yields a clean *Anacrusis* barcode when the same leg extracts were sequenced again, it became obvious that “*Anacrusis nephrodes*” is at least five species and “*Anacrusis aulaeodes*” is at least two species. However, all seven barcode-defined taxa appear to be just as much generalist as is the collective whole represented by two morphologically-defined species. These “true” generalists co-occur with many tens of species of other Tortricidae that range across many degrees of food-plant specialization, from extremely species-specific (e.g., *Pseudatteria volcanica* feeding on just three species of *Mollinedia* (Monimiaceae) and *Sparganocosma janzeni* (which became three when barcoded) feeding on just *Asplundia utilis* and *Carludovica costaricensis* (Cyclanthaceae), to the extreme generalist *Anacrusis* described above. When the time comes to ask the how and why questions of these consumers of a great diversity of truly nasty plant defenses, the parataxonomists are the obvious team, already on-site and familiar with the caterpillars and their food plants, to conduct all of the field work to whatever degree the budget permits.

### 3) Accuracy of food plant identification in the field at the start of the information chain

How does one “know” that the food plant species for a given caterpillar was correctly and accurately recorded by the parataxonomists? They are dealing with a very large flora of 1,000–3,000 species of food plants in any portion of ACG, and food plants are usually sterile and often juvenile at the time when a caterpillar is found. Food plant data is often critical for later attaching species-level significance to the ACG groups of barcodes in an NJ tree of morphologically “identical” moth specimens. For example, if the rain forest *Asturodes* fimbriauralisDHJ02 (Crambidae) is found to eat only *Colubrina* (Rhamnaceae) and the fully sympatric *Asturodes* fimbriauralisDHJ01 and *A.* fimbriauralisDHJ03 eat only *Gouania* (Rhamnaceae), the analysis wants to be 100% certain that the plants were correctly identified at the time of caterpillar collection.

Food plant identification by the parataxonomists is a multi-way iterative integrative process, and there are many moving parts.

- The parataxonomists come to know the species of plants not by keys, courses or published field guides (there are none for ACG plants as a whole), but by first noticing what appears morphologically to be a species of plant on which they have found a caterpillar. They then have to return to that species repeatedly to obtain fresh food (they change the caterpillar food every 3–5 days, depending on the weather), and in hopes of obtaining more of the same species of caterpillar (both for taxonomic confirmation and to get parasitoids). Caterpillar rearing requires remembering not only what the plant and caterpillar look like, but exactly where the collection happened – which individual plant at what curve of which trail – and building a mental map of where the accessible individuals of different plant species occur. While this creates a very heterogeneous and one-species-at-a-time taxonomic knowledge of the plants, it also means that the plant species in that place at that time is familiar as a living set of organisms, rather than a wobbly match to key characters defined from a herbarium or “the literature”. The plant species is therefore first baptized by the parataxonomists with a homemade common (and usually interim) name and plant collection voucher code (when first encountered as an inventory food plant, every unfamiliar plant species is classically herbarium-specimen collected, sterile or otherwise).
- Any given ACG parataxonomist works largely at a specific rearing barn/biological station for many years and all seasons, and repeatedly collects the same species of caterpillars from the same species of plants (and sometimes the same individuals). Multiple rearings over many years are not only for confirmation of host-specificity, but also to document the pool of parasitoids and to eventually link photographs of long gone/dead unknown specimens with a successful rearing years later. This is a self-correcting and fine-tuning process, reinforced by the failed rearings when a mistake is made and the caterpillar dies of starvation.
- The adult moths and butterflies from every rearing are checked first at the time of eclosion from the pupa, by the parataxonomists themselves, against the food plant species that was initially recorded at the time of caterpillar capture, and then again by Janzen a few months later. Discordances and apparent discrepancies are then checked by inspection of plant remains in rearing bags and bottles (which are saved for at least a year after eclosion). This quality control step usually distinguishes between the occasional identification errors and the rare (but real) ovipositional “error” (variation) and subsequent survival of a caterpillar on its “not usual” food plant.
- The plant species itself eventually receives a formal scientific name by (often) being already known, even in its sterile stages, by one of the three experienced ACG plant parataxonomists. They circulate among the biological stations while conducting the ACG plant inventory (both classically and through DNA barcoding), and more rarely, require further collecting of reproductive stages at later times by the parataxonomists. Clasical herbarium specimens are also deposited in INBio and discussed with other plant taxonomists. Since ACG plant barcoding was only begun in 2008 [33], it is only now becoming apparent that a few of the ACG food plant species are also made up of complexes. This has not, to date created ecological confusion because at least within ACG, the species pairs are microgeographically parapatric and the food plant records are then being retroactively upgraded. For example, the common vine *Vachellia tenuifolia* (formerly known as *Acacia tenuifolia*; Fabaceae) has a dry forest barcode-defined population and a rain forest barcode-defined population, with a pair of cryptic sibling species of *Urbanus* Hesperiidae to match. The formal scientific name is eventually learned by the parataxonomists on a one-by-one basis, and then retroactively replaces the interim names in the previous database records, rendering the subsequent Lepidoptera barcode collateral yet more universally useable.
- The parataxonomists then come to self-corroborate and know higher taxon groupings by the accumulation of examples within them. Bignoniaceae are therefore not known to them by the family-level key characters used in a herbarium circumstance, but rather by the (largely vegetative) gestalt of the collective array of members of that family occurring in the vicinity of a given rearing barn/biological station and on which have been found caterpillars. The caterpillars and their barcodes become another piece of correlative information to triangulate on whether a new (to them) adult moth or butterfly has been correctly attributed to its correct food plant when it was a caterpillar. The inventory becomes a light rain of sentences like “hey, that was probably not the correct food plant identification for that plant because spilomeline Crambidae have not been found (so far) feeding on *Croton* (Euphorbiaceae) in thousands of rearing records.” At this time, the parataxonomist and Janzen ask 1) was there a mistake in reading the voucher code off the rearing bottle or bag (re-examine the plant and/or pupal remains in the container), or 2) was there an error in the specimen handling in the sampling-barcoding process (match the morphological specimen against its barcode), or 3) does the usual food plant for this spilomeline moth just happen to have a leaf that looks superficially like that of one of the species of ACG *Croton*. If the latter, the parataxonomist may go back to where the caterpillar was found and return with a decision. All of this effort means that when caterpillar barcode, caterpillar/adult morphology, and food plant do not seem to match as expected, the inventory eventually sorts it out by working backwards towards the base of the information chain (or in the worst rare case, discards an unresolvable record). However, it is done, having the insect barcode (and hopefully shortly, the plant barcode) for identifcation triangulation has greatly increased the accuracy of the plant identifications.

ACG plant barcodes themselves are anticipated to play an enormously important role in the future in connecting future food plant records with those being recorded today by the parataxonomists. These staff members will eventually retire or move elsewhere. For their replacement parataxonomists to learn how to field identify these thousands of the same species of plants one-by-one and largely sterile will be a daunting, slow and inaccuracy-riddled process that will be greatly improved by having plant barcoding capacity in the field. It is one (quite possible) thing to know that this set of plants with these vegetative traits is one species, and quite another to accurately connect that field understanding to the understanding that another person had decades earlier for the same species of plant.

### 4) Iterative feedback from barcoding results to the parataxonomists

The first and later NJ trees received from the sequencing process through BOLD (http://www.boldsystems.org/views/login.php; [34]) are used in two different directions by us acting as a centralized clearing house both in the field and at the University of Pennsylvania, and during museum visits. We go downstream to fine-tune identifications in the primary project databases, and simultaneously alert collaborating taxonomists as to apparent mis-IDs and the presence of multiple barcode clusters within what was thought to be one morphological (or food-plant-eating) biological entity (with each barcode cluster potentially being a previously unrecognized species). This creates interactions that improve the quality of the raw data and subsequent analyses, but also creates more alpha-level and curational work and stress for the taxasphere. These interactions are not the focus here [see 3].

Moving this information upstream, back to the parataxonomists and the field inventory activity, creates more work for the inventory structure but simultaneously strengthens its taxonomic and ecological accuracy. We illustrate this with some examples of frequently repeated scenarios.

- The parataxonomists have to absorb and work with the consequences of relatively frequent name changes, the impossible-to-recognize-in-the-field cryptic species, and the increasing uncertainties in identities generated by barcoding (as well as by other more traditional dynamic processes within the taxasphere). For example, the caterpillar of the distinctive large butterfly “*Prepona laertes*” (Nymphalidae), now termed “*Prepona demodice*” rather than “*Prepona laertes demodice*”(see Figure 3 in [3] and cover image for that issue), was found and reared by the inventory first in 1979 while eating Fabaceae, and then a few times per year subsequently. In 1982 it was found eating Chrysobalanaceae. It was dutifully recorded as being a two-family-eating species that has been “done” by the inventory. In 2004, by which time the parataxonomists knew both adults and caterpillars well by their scientific name, 5 voucher specimens were routinely barcoded and found to display a deeply separated pair of barcode clusters that were then reinforced with more samples. One cluster (n = 45; “*Prepona* demodiceDHJ02”) was found to match perfectly with caterpillars found feeding on Fabaceae and the other (n = 22; “*Prepona* demodiceDHJ01”) with caterpillars found feeding on Chrysobalanaceae, in both ACG dry forest and rain forest. The solitary caterpillars are mimics of dead leaves and located serendipitously by searching whole treelet crowns 1–4 m above the ground. It has taken the parataxonomists 31 years to find and successfully rear a sample of this magnitude. This sample is a taxonomic module within a total of about 6,800 look-alike inventoried nymphalid caterpillars of about 35 species (*Memphis*, *Archaeoprepona*, *Agrias*, *Siderone*, *Consul*, *Zaretis*) that are sympatric with it while feeding on about 50 species of plants. The parataxonomists now know both species by their newly learned interim names, and continue to find, record and rear them for their contained parasitoids, and but they have to use the food plants as the key character, with all eclosing adults (to date) barcoded many months later for confirmation. Fragments of cadavers from parasitized caterpillars or rearing failures could also be barcoded at serious expense, but is not deemed to be necessary owing to the perfect match of the adult barcodes to the two very different food plant families.
- It was initially assumed that the inventory would remain manageable by restricting the kinds of collateral data to be gathered about each caterpillar, and therefore each species, as well as restricting the inventory to the 1,200 terrestrial km^2^ of ACG. An ACG caterpillar would be found, photographed, identified with a stable scientific or interim name, databased, displayed on the web (along with its adult), and declared arbitrarily to be “done”. However, the parataxonomists have now had to shift into a different paradigm, where the former protocol is operational, but the barcoding has revealed that many “done” species are indeed complexes that need more sampling and have more complicated (and less stable) names. Additionally, many more samples need to be (re)collected and reared in order to ecologically, morphologically, and/or microgeographically puzzle out species boundaries.

And this additional level of complexity adds further instability to the parataxonomist's challenge of learning names and what they mean. Parataxonomists learn not only how to distinguish plant and caterpillar species in their complex habitats by appearance or ecology, but also learn to refer to these by their polysyllabic Latin taxon names (spelled more or less correctly). Like all users of taxonomy, parataxonomists are thrown off balance when carefully-learned names change, or they are split up for any reason (taxonomic revision, barcoding discoveries). When barcoding reveals that a given species is a complex of cryptic species, the old familiar name can only apply to one or none of these. Furthermore the presence of barcode splits emphasizes that it is not as ecologically or microgeographically as widespread a species as was thought. Consequently, it is often the case that it is not clear whether any of the ACG cryptic species match the holotype and therefore deserve the name, and if so which one (see [35] for an example of four extremely similar species of *Perichares* skipper butterflies (Hesperiidae), one of which apparently matches the holotype while the other three were unnoticed and undescribed). Which, if any, of the two barcode-recognized species of ACG *Cocytius lucifer* (a huge sphingid moth, see Figure 3 in [3]) matches the holotype of *Cocytius lucifer* from the Yucatan Peninsula? One ACG species is apparently resident in the ACG rain forest and the other apparently migrates seasonally back and forth between the dry and rain forest.

**Figure 5:**
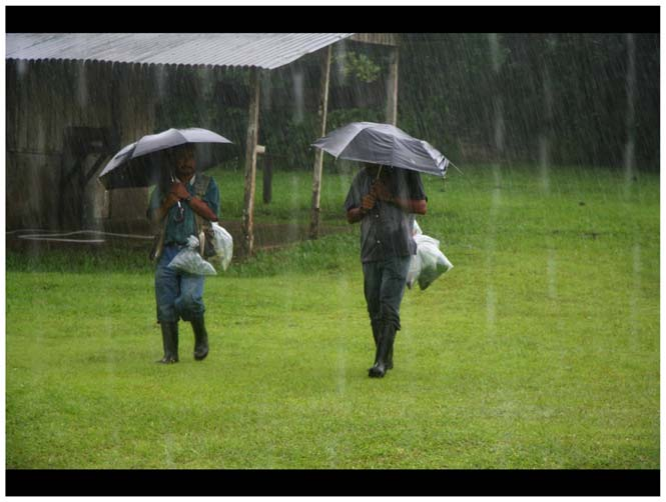
These two parataxonomists (“gusaneros” in the vernacular) – Calixto Moraga and Manuel Ríos - at Estación Biológica Pitilla are returning to the caterpillar rearing barn from a rain forest search, with their victims in bags with their food plants and bearing magnificent shelter from the elements. (2010). Image credit, Daniel Janzen.

If we cannot know what name goes with what in our well-outfitted urban laboratories, we cannot expect the parataxonomist to know in the beam of a flashlight in a rain forest rainstorm (Figure 5). At the field level, they learn that certain species have become very interesting and are specially targeted for search. Pragmatically, they continue to use the earlier aggregate name through the rearing process until tissues are barcoded and collateral data are compared. This avoids the case of the parataxonomist submitting the record as *Cocytius* luciferDHJ01 and getting it back as *Cocytius* luciferDHJ02, thereby thinking that he or she has “made a mistake”, something that the parataxonomists are particularly proud of doing only very rarely. It has been found to be better to initially record the caterpillars as simply *Cocytius lucifer*, unless the specimens are from a place or ecology in ACG where only one of the cryptic segregates is firmly believed to be resident.

### 5) Databasing errors

While in the beginning (1978) all data was recorded in field notebooks, by about 2000 all records were made in the field directly into FileMaker Pro databases. Each rearing barn and biological station has its own Apple laptop and is completely in charge of its own database for and during that year. Fusion into the main project database occurs at the end of the year, following compatibility checks for collateral data (place names, insect and plant names, date structure, etc.). The FileMaker Pro simple flatfile structure with the expected fields (in many ways the same fields as in any Darwin Core array of fields documenting a museum specimen) is far more than an information storage device. The individual fields in the record partitions the task of handling a mountain of species-specific detail into many small (and therefore manageable) one-at-a-time successive short stories. The barcodes and barcode-needed information, added from 2003 onward, and the information/analysis feedback from the barcodes, is just another set of fields to tag onto the sample, in fact or in memory. However, the database itself allows accurate on-the-spot real-time summarization of what was recorded about that species-level taxon in that year and in all previous years. When barcoding was added to the inventory, it definitely made the parataxonomists yet more aware of sibling species and what they imply, now that they can “see” them through the lens of barcoding, albeit sometimes after a long delay after the specimen collection date. Barcoding also provided a very real window into phylogeny, the process of evolution, and the role and messages of DNA in the field biology of “their” caterpillars, food plants, and parasitoids. Conducting science-based biodiversity inventory is an agile and straightforward way of experientially leading those lacking formal education into the predictive power of integrating scientific thinking into daily life. Adding DNA barcoding has conspicuously improved the process.

**Figure 6 pone-0018123-g006:**
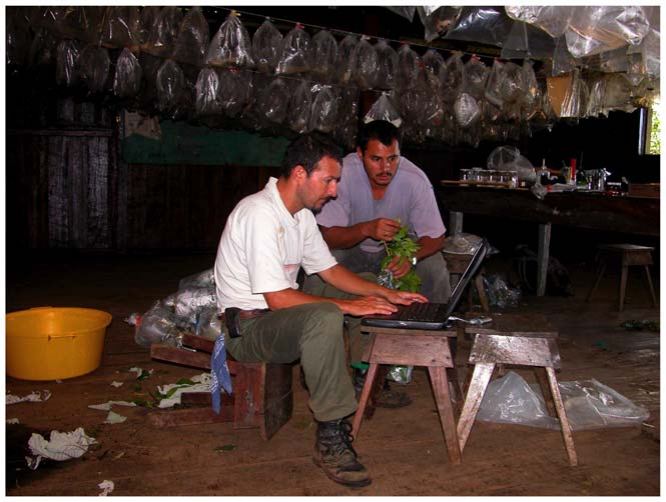
These two parataxonomists (Freddy Quesada and Harry Ramirez of Estación Biológica Cacao) are doing routine data entry in the rearing barn on the day of caterpillar collection. Plastic bags hanging in the background contain pupae waiting to eclose, while caterpillars in their bags with fresh foliage are outside in indirect sunlight. Harry is ensuring that Freddy enters the correct species name for the food plant in the record they are constructing (February 2003). Image credit, Daniel Janzen.

Prior to adding DNA barcoding to the inventory, the parataxonomists were already dealing on a daily basis with an enormous complexity of name- tagged data bits (Lepidoptera, parasitoid flies, and wasps, food plant names at the family, genus and species levels). Many of these bits are peculiar to the particular successional and ecosystem characteristics of the vicinity of their respective rearing barns and biological stations. All data is entered (Figure 6) into a 60-field flat file database record for each individual caterpillar, to sum to about 40,000 records/year for the entire project. There are about 20 fields for the initial record, followed by more at later times, added by the parataxonomists and other information processers.

There are three kinds of occasional errors in data entry, none of which are directly made more difficult by barcoding, but the last two below can quite easily impact barcoding analysis and results further down the processing chain. Any and all are greatly benefitted by iterative updating of the inventory record in BOLD before the sequences and their collateral can freely move into the public domain, GenBank or other aggregators.

There are occasional misspellings of place names, taxon names (interim or otherwise) and prose (in the comment fields). These errors are easily corrected through a variety of internal checks, though if persisting can cause confusion much later if a misspelling is also an actual other name. *Archaeoprepona demophoon* is easy to confound with *Archaeoprepona demophon* in the field (both are large Nymphalidae with deceptively similar yet quite different caterpillars and adults) unless it is realized that they almost never share food plants. There are three rivers with the name “Rio Negro” in ACG, and “Rio Gongora” is a very different place than “Cerro Gongora”. However, errors caused by such within-project similarities also become well known and are searched for explicitly, and when new sites are baptized, a major effort is made to avoid synonyms and look-alike place names.

There is occasional application of the wrong name to a taxon (food plant or insect). This may be due to mental lapse, confusion, carelessness, and most frequently of all, by duplicating a record (for efficient data entry) and then failing to update the appropriate nomenclatorial fields in the copy when entering data for the next specimen. Usually the substitute name is wildly inappropriate and immediately reparable, such as when this ludicrous name appears in a summary list. It may be particularly visible in an NJ tree and has been tagged onto a perfectly good barcode for another species (occasionally, though the name is correct but the discordant barcode is due to contamination). This is usually confirmed and corrected by comparing the other field contents and with those of the record before in the succession of database entries: “no, *Croton schiediana* is not in the Fabaceae and its caterpillars have nothing in common with caterpillars that eat Fabaceae, but the food plant of the previous record is indeed in the Fabaceae”. In this project, we have chosen not to use pick-lists for taxonomic names because it is too easy – and irretrievable- to select an incorrect neighboring choice by accident. A misspelling is much easier to notice and to correct, even though direct typing and database corrections take longer.

A second kind of barcode-impacting “misidentification” occurs when the parataxonomist applies a best-guess but erroneous (though valid) name to a specimen (usually a caterpillar or pupa) in the field at the time of collection, and because it is a “reasonable” name, it is not noticed to be in error until the actual barcode sequence appears in an unexpected barcode cluster in an NJ tree. At that time, however, through the usual iterative process of using NJ trees to sort specimens, a corroboration match is made with the photograph of the adult that was taken at the time of de-legging, and the confusion eliminated.

There are transpositions, deletions, or just plain typographical errors within a few digits in voucher codes (or, more rarely, dates – particularily in the first month of the year) and in counts of specimens. While this happens rarely, it is among the greatest causes of headaches during analysis of barcode results and correlations with collateral data. In this case, a barcode sequence receives the collateral, including the name, that belongs to a different record. The resulting nonsense in the NJ tree requires that the physical specimen be found and examined in comparison with these results and the database record. The need to be able to correct such errors is a major reason for wanting the voucher specimens to remain within reach until such errors have been purged from the data stream (usually a 2–6 month process). Occasionally the record cannot be recovered because the error cannot be puzzled out. In this case, the entire record, specimen and barcode information is best discarded.

On the other hand, what appears to be a numerical error can also be a signal of a contamination in the sampling process (especially with older specimens of scale-covered Lepidoptera if the leg-plucking forceps were not thoroughly cleaned) or in the sequencing laboratory itself.

The parataxonomists catch many numerical errors and typos themselves, but errors and typos also are noticed in the Santa Rosa clearing center when we compare the frozen adult (as newly delivered) against the field records at the time of deciding the fate of that specimen (preserved in alcohol, museum-quality spread, beetle-pinned, discarded, etc.). Within several months we pass computerized feedback to the parataxonomists' within-year versions of the rearing barn databases as to what was the fate of the specimen. At that time we may also ask that more focus be put on that species because, for example, we are beginning to suspect that there are cryptic species as exposed by the preliminary barcode results.

### 6) Contrast of BioLep parataxonomists with caterpillar-rearing parataxonomists in the ACG inventory

In 2006, after 3 years of intense retroactive barcoding of the rearing inventory vouchers stored since the ACG inventory began in 1978, as well as barcoding the annual incoming stream of new voucher specimens, it became clear that the construction of a total barcode library (directory) for the ACG Lepidoptera would of course require as long as the future decades of caterpillar inventory. However, BIO (Biodiversity Institute of Ontario, the home of the barcoding initiative at the University of Guelph), in the role of forerunner of iBOL (http://ibol.org/), offered to speed the process by barcoding vouchers of all the species of wild-caught adult ACG Lepidoptera that the inventory could sample as well. Such an inventory is therefore a repeat of the intense census of ACG adult Lepidoptera that we conducted between 1978 and 1993. The hundreds of thousands of specimens from this previous inventory are deposited in INBio in the outskirts of San Jose, Costa Rica (http://www.inbio.ac.cr), and generally too old to inexpensively yield high quality full-length DNA barcodes (BIO is, however, presently developing protocols for harvest of such aged within-museum barcodes).

**Figure 7 pone-0018123-g007:**
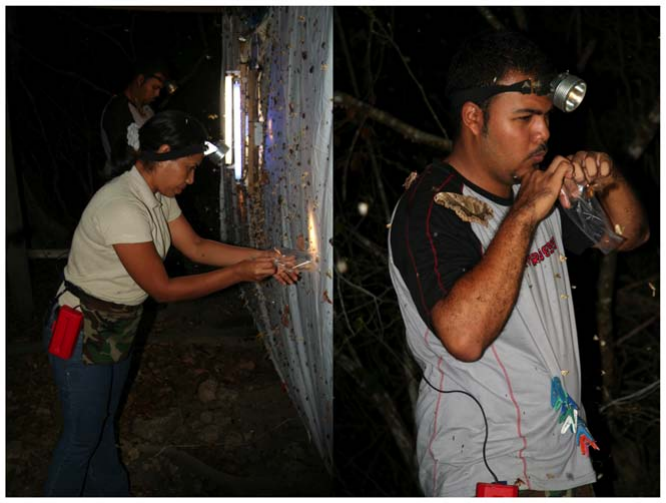
These two BioLep parataxonomists (Hazel Cambronero and Sergio Ríos) are collecting at a car-battery-powered light (Sector Santa Rosa) in order to construct the adult Lepidoptera ACG barcode library. Each moth is collected individually into a small used-only-once plastic bag to avoid contamination with the scales from other moths, and then frozen, later to be sorted while still in the bag, before spreading and drying for subsequent de-legging for barcoding. More than 4,000 species of moths have been collected from this particular light in the three decades of moth inventory of ACG. (June 2006). Image credit, Daniel Janzen.

Consequently, despite having a reasonable idea of how many thousands of species of Lepidoptera live in ACG, in 2006 we initiated the ACG BioLep project to conduct a sort of bioblitz of the ACG adult Lepidoptera fauna for the express purpose of building the barcode library much faster than could be done by relying entirely on the adults produced by the caterpillar inventory. Two very experienced caterpillar parataxonomists (“gusaneros” in local vocabulary), Ruth Franco and Freddy Quesada, were “retooled” into this kind of adult inventory. They trained two new parataxonomists, Hazel Cambronero (housewife) and Sergio Ríos (former taxi driver), and the four began an intense roving moth inventory with blacklights (e.g., Figure 7), to be later followed by collecting with nets. As expected, this process now generates another quite different array of about 18,000 barcodes/year. For medium-sized to small moths, there is to date only modest overlap with the barcodes created by caterpillar rearing. The largest reason for the modesty of overlap is that many species of ACG moths only rarely (if ever) go to lights hung out in the forest, and a 4-member team is not a large enough operation to thoroughly sample (as yet) all the ACG habitats and ecosystems that the caterpillar inventory has been searching for 30 years. The two efforts in parallel will eventually converge on an inventory list in common, though the technical incompleteness of both (and other) survey methods will always mean that simultaneous application of a variety of methods is required to even approach a true total Lepidoptera inventory. At present (early 2011), the caterpillar inventory has reared and barcoded about 5,000 species and the BioLep inventory about 7,000 species, for a combined total of about 9,000 barcode-distinctive species. This result has in turn elevated the estimated ACG total from 12,500 species to 15,000 species.

The BioLep adult barcoding immediately encounters the same taxonomic problem as does classical adult Lepidoptera inventory in any place, since there are only two groups of datapoints: there are those that are morphology-based and those that are barcode-based (with a smattering of microgeography tossed into the mix). While the barcodes are superb for associating sexes of highly sexually dimorphic species (e.g., the *Anacrusis* example cited above and [32]), when a morphology-based “species” collected from lights displays two or more groups of barcodes (a commonplace), about the only avenue left for species discovery is moving to other genes and/or more detailed scrutiny of morphology. The latter sometimes “works” but not always, and requires substantially more finances to support both the gene-based and the morphology-based taxonomy. For the ACG inventory, all of the BioLep budget has come from private donations to the Guanacaste Dry Forest Conservation Fund (http://www.gdfcf.org) and the Biodiversity Institute of Ontario (BIO), by donors willing to support parataxonomists and DNA barcoding.

The practical outcome is that the BioLep team is frequently told to “collect and send for barcoding a sample of all individuals of species such and such”, because we now know that it is apparently a complex of cryptic species, and we need all possible specimens so as to attempt to resolve what is happening. Another outcome, as illustrated by “*Adhemarius gannascus*” and “*Xylophanes porcus*”, two common large sphingid moths in ACG light traps, is that the BioLep team is told to collect all that arrive at the lights because we now know each of these two “species” are 3–4 slightly microparapatric species and only by barcoding can we reliably identify them and work out their distributions within ACG.

**Figure 8 pone-0018123-g008:**
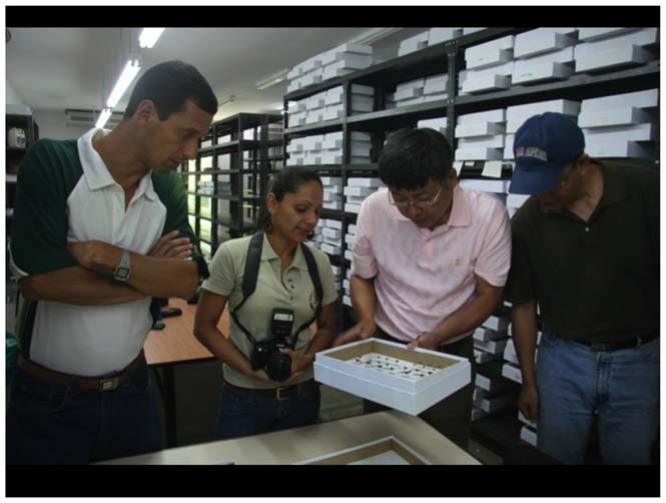
Ruth Franco, a BioLep parataxonomist, is explaining DNA barcoding of ACG Lepidoptera to the University of Costa Rica (left, Dr. Gustavo Gutierrez) and the university (center, Dr. Xing-Jie Liang) and government community (right, Dr. Jia Shangang) of the Peoples' Republic of China. The white specimen boxes (see Figure 4) in the background are filled with thousands of barcoded vouchers of moths and butterflies that were collected and barcoded by Ruth and her three parataxonomist teammates. (September 2009, BioLep Building, Estación Biológica, Sector Santa Rosa). Image credit, Roger Blanco.

**Figure 9:**
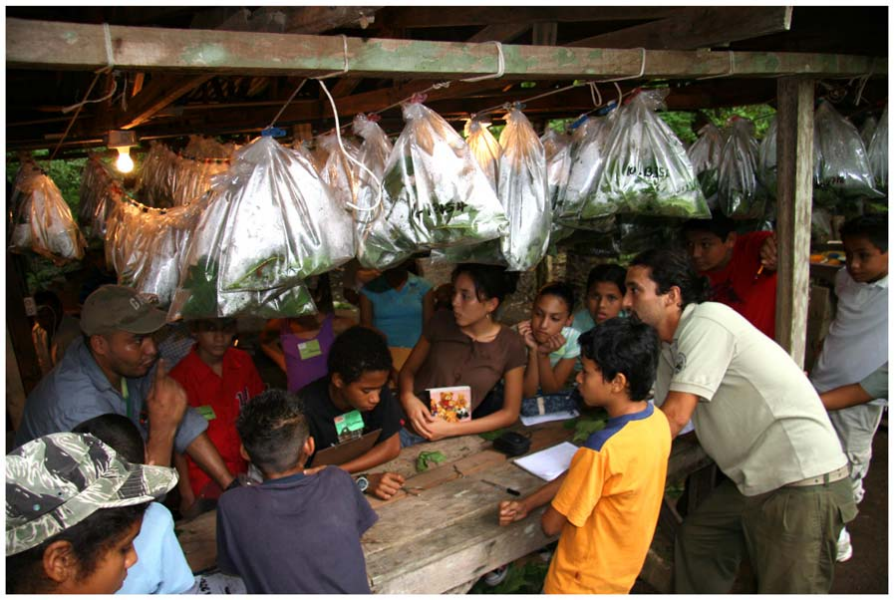
This parataxonomist (left, blue shirt, gray hat, Johan Vargas) is describing caterpillars [**8]**, [16****] and caterpillar barcoding genetics to an ACG teacher (right, beige ACG shirt, Alban Jimenez) in the ACG Programa de Educación Biológica (PEB) at the Santa Rosa rearing barn during a special weekend reward course for outstanding 4–6^th^-graders from neighboring schools. (June 2010). Image credit, Pablo Vasquez.

The BioLep team has serendipitously – owing to being based at the Estación Biológica Santa Rosa in the ACG administration area – proven to be a priceless resource for explaining DNA barcoding and all that it portends to adult and children visitors (e.g., Figure 8), and explaining the ACG adult and caterpillar-food plant-parasitoid inventories (e.g., Figure 9). We have found that the on-site explanation far outweighs published descriptions of the process for absorbing and understanding. And, simultaneously, this process inconspicuously demonstrates the biodiversity development of human resources from this rural zone as an active and on-going process of biodiversity conservation and poverty alleviation through intellect-based, as well as field-hardy, local employment [e.g.], [ Janzen 8]–[12], [ 21]–[23], [ 36].

## Discussion

Members of the university-educated community, both internationally and in-country, sometimes doubt that members of the rural workforce with minimal formal education can conduct a complex activity like this in the field, with little or no direct supervision, and with the primary feedback being their own discovery and rearing results, coupled with the accumulated information in their databases. The inventory of ACG Lepidoptera and its included subproject of DNA barcoding this biota demonstrates otherwise.

It would be negligent and human-resource wasteful to conduct an inventory of thousands of species within a higher taxon in a large complex tropical area without having the fieldwork conducted by a team of career parataxonomists working in concert with the taxasphere. This combination not only gets the job done, it simultaneously embeds the process, and biodiversity discoveries and awareness, in the resident neighboring population. It is both startling and revealing, when upon walking into a rural village grocery store (with a per family cash income of less than $3,000/year), the owner looks up from behind the counter and says “Do you know your web site is down?” And then complains “How can my daughter do her homework if she can't get into your web site?” Equally, it would be negligent to contemplate such a project without routinely barcoding just about everything, both to discover the biodiversity that cannot be easily recognized without barcoding (whether initially or later), and to guide and corroborate the daily identification process in both the inventory and biodiversity management of the conserved wildland.

## Methods

As corresponding author, I confirm to the best of my knowledge that all people in Figures 3, 5–9 have agreed to the inclusion of this photo in our paper.
